# Macrophages of the “Heart-Kidney” Axis: Their Dynamics and Correlations with Clinical Data and Outcomes in Patients with Myocardial Infarction

**DOI:** 10.3390/jpm12020127

**Published:** 2022-01-18

**Authors:** Maria Kercheva, Vyacheslav Ryabov, Aleksandra Gombozhapova, Maria Rebenkova, Julia Kzhyshkowska

**Affiliations:** 1 Central Research Laboratory, Siberian State Medical University, 2 Moscovsky trakt, 634055 Tomsk, Russia; rvvt@cardio-tomsk.ru (V.R.); gombozhapova@gmail.com (A.G.); 2 Cardiology Research Institute, Tomsk National Research Medical Center, Russian Academy of Sciences, 111a Kievskaya Street, 634012 Tomsk, Russia; mariambf@mail.ru; 3 Laboratory of Translational and Cellular Biomedicine, National Research Tomsk State University, 36 Lenin Avenue, 634050 Tomsk, Russia; 4 Department for Innate Immunity and Tolerance, Institute of Transfusion Medicine and Immunology, University of Heidelberg, 1-3 Theodor-Kutzer Ufer, 68167 Mannheim, Germany; julia.kzhyshkowska@googlemail.com

**Keywords:** myocardial infarction, cardiac remodeling, cardiac macrophages, kidney macrophages, CD68, CD206, CD163, stabilin-1, inflammation

## Abstract

Changes in the macrophage infiltration of kidneys in rodents under ischemic conditions may affect cardiac macrophages and lead to development of adaptive cardiac remodeling. The aim of our study was to translate experimental findings into clinically relevant applications and assess the features of macrophage infiltration of the kidney and its correlations with changes in macrophage infiltration of the myocardium and with clinical data in patients who experienced a fatal myocardial infarction (MI). We examined fragments of both organs taken from patients (*n* = 30) who suffered from fatal MI. Macrophage infiltration was assessed by immunohistochemistry. Macrophage infiltration of the kidneys in patients with fatal MI is heterogeneous. The early period of MI was shown to be characterized by the prevalence of CD163+ and CD68+ cells, and in the long-term period by only CD163+ cells. However, only the level of CD206+ cells in the kidneys showed the dynamics representing the late MI period. Its decrease accompanied increase in the numbers of cardiac CD68+, CD163+, CD206+, and stabilin-1+ cells in the infarct area. Kidney CD206+ cells had more correlations with cardiac macrophages than other cells, and the presence of these cells also correlated with impairment of renal function and early death.

## 1. Introduction

Acute kidney injury (AKI) accompanies myocardial infarction (MI) in every fifth patient [1]. The presence of kidney damage is associated with poor short- and long-term prognosis and outcomes after MI and a high incidence of recurrent MI, heart failure (HF) and progression of chronic kidney disease (CKD) [2,3,4]. The body’s response to ischemia is not limited to local myocardial injury. Excitation of the sympathetic nervous system (SNS) occurs under ischemic conditions and leads to the activation of two systems: (1) the renin-angiotensin-aldosterone system (RAAS) and (2) macrophages of the “heart-kidney” axis [5]. A large amount of data has been accumulated regarding interorgan interactions in the RAAS [5]. However, a lack of knowledge about the cellular−molecular basis of the cardiorenal relationship based on changes in the macrophages of the “heart−kidney” axis exists [6,7].

Experimental animal studies have shown that continuous conditions of ischemic sympathetic stimulation of renal collecting duct cells promotes the release by kidney macrophages of granulocyte-macrophage colony-stimulating factor (GM-CSF) into the bloodstream [7]. This cytokine activates the paracrine signaling pathway in the heart and induces polarization of inflammatory M1 macrophages into anti-inflammatory M2 macrophages [6]. This process causes the development of adaptive myocardial hypertrophy and cardiac fibrosis [7]. However, this mechanism of cardiorenal interaction has not yet been studied in humans. An in-depth study of the diversity of kidney macrophage phenotypes, their spatio-temporal relationship with cardiac macrophages, and adverse outcomes in patients with MI will permit us to determine specific phenotypes of kidney macrophages, which could become a target for intervention in future.

The aim of our study was to translate experimental findings into clinically relevant applications and to assess the features of kidney macrophage infiltration and its correlations with changes in macrophage infiltration of the myocardium and clinical data in patients who experienced a fatal MI.

## 2. Materials and Methods

The inclusion criterion of the study was a fatal MI 1 (*n* = 30). Myocardium (from the infarct (IA), peri-infarct (peri-IA), and non-infarct areas) and kidney fragments were obtained during autopsy and were used as materials for this study. The exclusion criteria included several parameters: (1) type II–V MI, (2) oncological disease, (3) infectious complications (sepsis, pneumonia), and (4) valvular diseases requiring surgical intervention. The study was approved by the Biomedical Ethics Committee of the Research Institute of Cardiology, Tomsk National Research Medical Center (Protocol No. 128) and was conducted in accordance with the principles of the Declaration of Helsinki. Autopsies were performed in accordance with the order of the Ministry of Health of the Russian Federation No. 354n dated 6 June 2013. Signed informed consent from a patient was not needed, which did not contradict the principles for conducting the study according to the Declaration of Helsinki (“informed consent”, para. 32).

Autopsy was performed within 24 h after death. The collected material was fixed in 10% buffered formalin for a day, after which standard histological tracing and paraffin embedding were performed using the Thermo Scientific Excelsior ES tissue processor. The paraffin blocks were stored in an archive for 6 years. After that, microtome sections of the kidney and heart were cut using a Thermo Scientific Microm HM355S rotary microtome. From each paraffin block, 10 sections were cut for kidney fragments and 20 sections for myocardial fragments. After that, the material was applied onto glass coated with L-polylysine, two sections per glass.

Macrophage infiltration of the myocardium and kidney was assessed by two independent experts using immunohistochemistry performed using an automatic immunostainer. For macrophage immunophenotyping, mouse monoclonal antibodies were used to detect the total macrophage marker CD68 (Cell Marque, dilution 1:500, clone Kp-1), antibodies to the M2 macrophage marker CD163 (Cell Marque, dilution 1:50, clone 10D6), and CD206 (Santa Cruz, dilution 1:100, clone C-10), and additional antibodies to the M2 macrophage marker synthesized at the Department of Innate Immunity and Tolerance, Institute for Transfusion and Clinical Immunology, University of Heidelberg, Mannheim, Germany (dilution 1:1000) [8].

**Figure 1:**
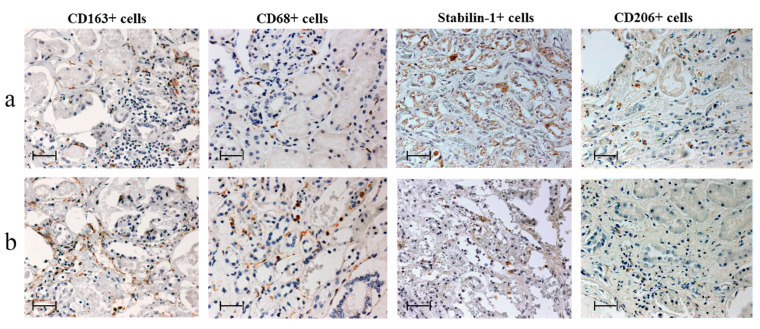
Changes in macrophage infiltration of kidneys according to the period of myocardial infarction (MI), immunohistochemistry, scale-bar 50 μm. **a**—early period of MI (*n* = 17), **b**—late period of MI (*n* = 13).

For visualization, the horseradishperoxidase-3,3’-diaminobenzidine, peroxidase-3,3’diaminobenzidine (HRP-DAB) system was employed. Immunohistochemical staining (Figure 1) was performed according to a standard protocol, as in our previous study with cardiac macrophages [8]. Two independent specialists used the Zeiss Axio Imager M2 microscope to count the number of macrophages in the myocardium and in the kidneys in 10 random fields of view (40× objective).

Patients were divided into two groups depending on the time of death after MI: (1) group 1 included those who died within the first three days from the onset of the disease and (2) group 2 included those who died within the period from day 4 to 28 [9]. Table 1 presents the clinical and anamnestic data on patients.

The resulting data were processed using the statistical STATISTICA 12.0 package. The quantitative data were tested for normality using the Shapiro–Wilk test. The age of patients and the level of creatinine upon hospital admission were described based on mean and standard deviation (M and SD, respectively), and other quantitative indicators that did not show normal distribution were described by the median and interquartile range (ME and Q1; Q3 respectively). Categorical indicators were described by frequencies and percentages. Quantitative indicators in the groups were compared using the Mann–Whitney test, and categorical indicators were compared using χ^2^ (Pearson’s test) and Fisher’s test. Correlations between the number of cells and clinical data were revealed using Spearman’s correlation coefficient. An r value (rank correlation coefficient) between 0.4 and 0.7 indicated a moderate correlation. Statistical hypotheses were tested with respect to a significance value of *p* = 0.05.

The multivariate logistic regression model included variables characterizing the onset of early death in the study group, such as a history of MI, ST-segment elevation MI (STEMI), anterior-inferior MI, presence of acute heart failure (AHF), functional class (FC) > I upon admission, and the number of studied macrophage cells in the kidneys. This model was used to determine the characteristics with the highest predictive value toward the onset of early death in the study group.

## 3. Results

The immunohistochemistry of myocardial and kidney tissue in patients with fatal MI revealed the presence of types M1 and M1 macrophages in both organs.

The number of CD163+ and CD68+ cells predominated in the kidney (Table 2) and myocardial tissue in the early period of MI (*p* = 0.0001). In the late MI period, they predominated in the peri-IA and non-IA in myocardium, however, in kidney and myocardial tissue (IA) the number of CD68+ cells decreased more significantly, than the number of CD163+ cells (*p* = 0.001) but remained higher than other cells.

**Figure 2:**
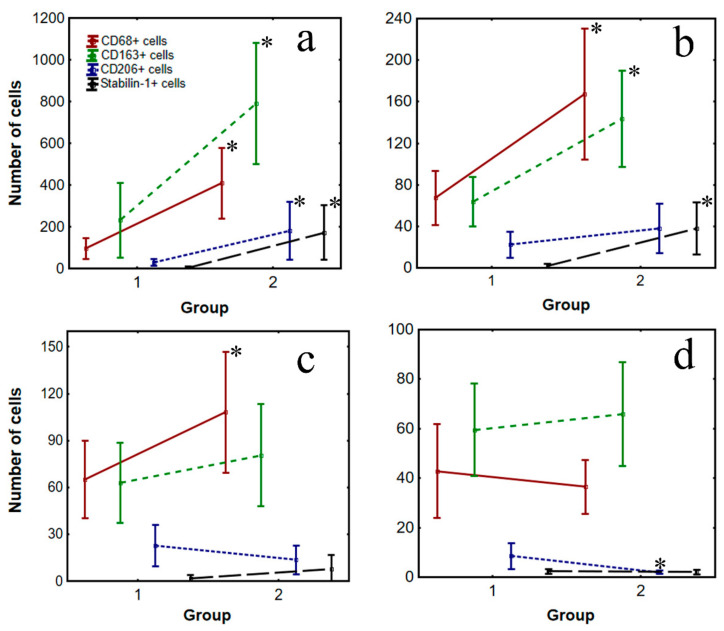
Dynamics of cardiac and kidney macrophages in patients (n = 30) with fatal MI. Note: **a**—infarct area of myocardium, **b**—peri-infarct area of myocardium, **c**—non-infarct of myocardium, **d**—kidney. ∗—statistically significant between the groups.

The number of CD206+ cells in the kidneys decreased by the late period (*p* = 0.0001); in the myocardium (IA), this number increased (*p* = 0.025). The number of CD68+ cells in all myocardial areas increased to the late period (*p* = 0.004); the number of CD163+ and stabilin-1+ cells also increased, but only in the IA and peri-IA of the myocardium (*p* = 0.005 and 0.001, respectively), as shown in Table 2 and Figure 2.

In addition, 10 patients from the study group had CKD in their medical histories. The presence of CKD was accompanied by a smaller number of CD206+ cells in the macrophage kidney infiltrate, and the numbers of other cells were comparable to patients without CKD (Table 3).

**Figure 3:**
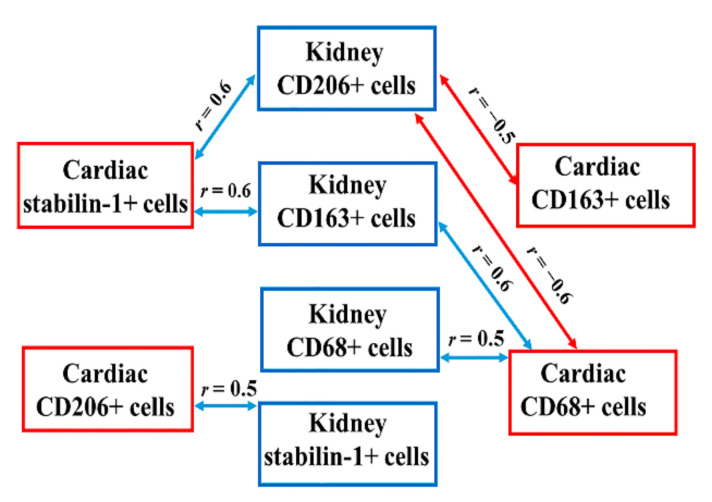
Correlations between kidney and cardiac macrophages in patients with fatal MI.

The spatio-temporal correlation between kidney and cardiac macrophages was then assessed (Figure 3).

Characteristics of MI, such as STEMI, anterior-inferior MI, history of MI, and AHF FC > I upon admission, which were associated with a fatal outcome in the study group, were included in the multiple regression model along with the number of investigated kidney macrophages (Table 4). As a result, along with characteristics such as history of MI, and AHF FC > I, the number of kidney CD206+ cells was associated with an early and fatal outcome (β = −0.4, *p* = 0.005).

## 4. Discussion

Previous results on the role of the innate immunity system in post-infarction myocardial regeneration in humans and animals [8,10,11] and its place in cardiorenal interactions in animals provided the rationale for studying this issue [6,7,12,13]. Involvement of monocytes/macrophages in both the inflammatory and regenerative phases of post-infarction myocardial remodeling has already been confirmed by the presence of their prolonged CD68+, CD163+, CD206+ and stabilin-1+ macrophage infiltration in both IA and non-IA of the myocardium in patients who experienced a fatal MI [10]. However, very little is known about the phenotypic characteristics and functions of resident macrophages in human kidneys [14,15,16]. The similarities and differences in resident macrophages in kidneys between rodent models and human kidneys have not yet been determined. Along with assessing macrophage infiltration of kidneys and its dynamics, our aim was to determine the presence and nature of the relationship between macrophage infiltration of the heart and kidneys, and its relationship with poor prognosis in patients with fatal MI.

The features of macrophage infiltration of kidneys in humans were first studied by Marshall in 1984 [17]. Kidney sections obtained during autopsy from patients with adenocarcinoma and nephroblastoma were assessed and compared with those of a control group consisting of patients who had died from fatal injuries. In addition, monocytes were also studied in the peripheral blood of patients with CKD and undertaking dialysis [14,15]. It was shown that the number of circulating classical monocytes CD14+ and CD16−, can predict the development of cardiovascular events and death in patients with CKD [12]. Experimental studies conducted with rodents showed that kidney macrophage polarization under ischemic conditions can affect cardiac macrophage polarization by activating the paracrine signaling pathway, which ultimately promotes the development of adaptive hypertrophy and fibrosis of the myocardium, which underlie its remodeling [7,18]. We were the first to study macrophage infiltration of the heart and kidneys in patients who experienced MI and to assess the dynamics of kidney and cardiac macrophages with respect to the periods of MI, and both inter-relationship and relationship with poor prognosis.

Macrophages are a pool of heterogeneous cells capable of rapid phenotype change under the impact of external signals arising from: (1) the microenvironment, (2) inflammation stages, and (3) exposure to cytokines and other signaling molecules [11]. This rapid change capacity is the basis for macrophage polarization, which is the capability of transition from one phenotype to another [19,20]. To date, many macrophage phenotypes [21] have been reported; however, the most used form of classification is dichotomous classification dividing macrophages into pro- (M1) and anti-inflammatory (M2) types [22]. For the analysis, we used M1 and M2 types of macrophages. Macrophage infiltration of the kidney is normally represented by resident M2 macrophages [23]. These cells are formed during embryogenesis [24], and their main function is homeostasis maintenance in the kidney, immunological regulation and regeneration of damaged renal tissue, and initiation of embryogenesis [25]. M2 kidney macrophages are maintained through self-renewal throughout life; however, in the damaged kidney, their reserves can be replenished from the pool of monocytes in the bone marrow [26], which probably occurred in our study and was demonstrated by a quantitative change in the macrophage composition of the kidney.

CD68+ and CD163+ cells were found to prevail in the cardiac tissue, both in the IA and peri-IA, in addition to in the renal tissue of patients who experienced an MI [8,9]. CD68 is a histochemical marker of the general population of macrophages and belongs to scavenger receptor class D [27,28]. CD68 performs the main functions of macrophages, such as absorption of apoptotic and damaged cells, and its involvement in atherogenesis has previously been determined [29]. This marker showed a certain predictive value in oncology [30]. In our case, the increased number of CD68+ cells in the early period of MI, both in the myocardium and in the kidneys, indicated the implementation of the main function of macrophages, namely, involvement in the systemic inflammatory response of the body to ischemic damage of tissues and organs. This finding was also confirmed by the observation that the high number of CD68+ cells in the kidney was found to be associated with their increased number in the myocardial tissue. A decrease in the number of these cells relative to the number of CD163+ cells in the late period of MI, both in the renal and in the myocardial tissue, apparently indicates M1 type. The ongoing infiltration of the myocardium with CD68+ cells reflects a prolonged inflammatory response associated with adverse outcomes in the study sample. In addition, CD163+ cells prevailed in tissues at all time intervals. These cells probably represent a pool of tissue M2 type macrophages and dendritic cells. CD163 belongs to scavenger receptor class I, which is highly expressed on macrophages of the red pulp of the spleen, bone marrow, liver, lungs, and some other tissues [31]. Its high expression on macrophages can be observed in tissues during acute and chronic inflammatory processes [31]. In addition, this marker has been applied in oncology [32,33]; however, the physiological role of soluble CD163 has not yet been determined.

Infiltration of cardiac and kidney tissue and its dynamics were assessed by stabilin-1+ cells (M2-type) [34]. Stabilin-1 is in scavenger receptor class H4, and is expressed on tissue macrophages and sinusoidal endotheliocytes of the spleen, liver, and lymph nodes [34,35]. Its expression increases during chronic inflammatory processes and tumorigenesis [33,34]. In the study sample, the number of stabilin-1+ cells in the early period of MI was minimal, both in cardiac and renal tissue; however, in the late period, this number increased significantly in the myocardium, which probably indicates its anti-inflammatory nature. The relationship between the number of stabilin-1+ cells in the IA and CD68+ cells in the kidney probably indicates the processes occurring during development of cardiorenal syndrome in rodents [6,7]. It was found that cells of the renal collecting ducts can be active participants in cardiorenal interactions, can control inflammation in the kidney, and can cause a reduction in damage to the renal parenchyma, including that under conditions of an MI [7]. Under hemodynamic stress conditions in rodents with transverse aortic narrowing, the expression of calcium-binding proteins by S100A8 and S100A9 podocytes promoted the differentiation of renal M2 macrophages into inflammatory M1 macrophages by stimulating the expression of tumor necrosis factor (TNF) by kidney macrophages, which promote the secretion of GM-CSF by endothelial cells. An increase in the concentration of GM-CSF in the plasma leads to induction of the proliferation of Ly6Clo macrophages, an analogue of human M2 macrophages, in the mouse heart thus triggering the hypertrophic paracrine pathway in the heart [35]. Under conditions of hemodynamic stress, suppressed expression of genes responsible for the secretion of S100A8 and S100A9 proteins, including proinflammatory transcription factor Klf5 (Klf5KO mice), causes a decrease in the adaptive response of the myocardium to hemodynamic overload, which was confirmed by dilatation of cardiac cavities and increase in mortality compared to mice with active gene expression. The heart mass and cross-sectional area of cardiac myocytes in Klf5KO mice did not increase, a finding that probably indicates the absence of the development of adaptive myocardial hypertrophy and cardiac fibrosis. However, plasma renin in these mice remained elevated for 28 days after aortic transverse constriction, which indicates activation of the renin–angiotensin–aldosterone system (RAAS), an inducer of cardiac hypertrophy and fibrosis. In this case, the absence of hypertrophy and fibrosis in mice probably indicates that a certain threshold of the plasma level of GM-CSF is required to activate RAAS-induced cardiac remodeling [12]. Interestingly, Ly6Clo transplantation into Klf5KO mice lead to a significant improvement in survival after transverse aortic narrowing, while transplantation of Ly6Chi, an analogue of M1 macrophages in humans, did not show this effect [7]. These findings may indirectly indicate the relevance of M2 macrophages for myocardial regeneration after an MI, the violation of the expansion of which contributes to the progression of adverse cardiac remodeling. This possibility, however, was not demonstrated by in vivo results in humans and requires further study [35,36].

The most interesting and promising factor for further analysis were CD206+ cells, the number of which was similar to those observed during polarization, which most directly reflected the processes occurring in the myocardium and kidneys in response to ischemia.

Interestingly, the increase in the number of CD206+ cells in the kidney was associated with their lower number in the myocardium; a similar relationship for M2 macrophages was revealed in experiments with rodents [6,7]. Depletion of the pool of resident macrophages in the early period of MI due to their polarization into M1 macrophages, together with the replenishment of renal tissue with alien M1 macrophages, promotes the release of GM-CSF into the bloodstream, which causes the polarization of myocardial macrophages into M2 type. These data are confirmed by the inverse relationship between the number of CD206+ cells in the kidney and the number of CD68+ and CD163+ cells in the IA of the myocardium. A multivariate analysis yielded interesting and contradictory data in which an increase in the number of CD206+ cells in the kidney, along with well-known markers of poor prognosis in patients who experienced an MI, was associated with an early and fatal outcome. Early untimely regeneration in addition to prolonged inflammation can have a harmful effect on patients with MI [25]. The activity and time intervals of the regenerative processes can apparently present specific characteristics. In the process of continuous damage, persistent infiltration of the kidney by M2 macrophages can lead to constant production of several growth factors that facilitate healing the wound, and what was initially triggered as a reparative mechanism can subsequently be unfavorable and cause irreversible fibrosis and progressive renal tissue destruction [25]. Evidence is available describing experimental models of chronic renal diseases, such as diabetic nephropathy in animals, in which macrophages cause a shift toward chronic activation of the M2 phenotype in later periods of the disease, which eventually leads to the development of glomerulosclerosis, interstitial tubular fibrosis and, ultimately, renal failure [37,38]. Perhaps this shift was the reason for the reduction in the amount of kidney CD206+ cells in our sample in patients with CKD. On the other hand, some evidence that the depletion of macrophages during repair hinders recovery has been reported, an event that possibly occurred in our sample and was demonstrated by the decrease in the number of kidney CD206+ cells in the late MI period [39]. However, it is possible that the increased number of kidney CD206+ cells are associated with an early and fatal outcome in MI. For a better understanding of the location of this type of cells, it is necessary to study the function and content of these type of cells in a larger sample.

## 5. Conclusions

Macrophage infiltration of the kidneys in patients who experienced a fatal MI is heterogeneous. The early period of MI was characterized by the prevalence of CD163+ and CD68+ cells, and the long-term period, by only CD163+ cells. However, only the level of CD206+ cells in kidney showed these dynamics in the late period of MI. The decrease in CD206+ cells was accompanied by an increase in the numbers of cardiac CD68+, CD163+, CD206+ and stabilin-1+ cells in the infarct area. Kidney CD206+ cells showed more correlations with cardiac macrophages compared to other cells, and their presence also correlated with impairment of renal function and early death.

## Figures and Tables

**Table 1:** Clinical and anamnestic characteristics of patients (*n* = 30).

Parameters	All Patients	Group 1	Group 2	*p*
Number of patients, *n*	30	17 (57%)	13 (43%)	
Age, years	74.8 ± 9.8	73 ± 9.3	77 ± 10.1	0.3
Male sex, *n* (%)	12 (39%)	7 (42%)	5 (38%)	0.9
STEMI, *n* (%)	26 (87%)	16 (94%)	10 (77%)	0.2
**Infarct area**				
Anterior MI, *n* (%)	10 (33%)	8 (47%)	2 (15%)	0.1
Inferior MI, *n* (%)	8 (27%)	3 (18%)	5 (38%)	0.3
Anterior-inferior MI, *n* (%)	12 (40%)	6 (35%)	6 (46%)	0.6
**CAD risk factors**				
Diabetes, *n* (%)	9 (30%)	4 (23%)	5 (38%)	0.5
Arterial hypertension, *n* (%)	30 (100%)	17 (100%)	13 (100%)	0.4
Obesity, *n* (%)	10 (33%)	6 (35%)	4 (31%)	0.8
Hypercholesterolemia, *n* (%)	8 (27%)	2 (12%)	6 (46%)	0.9
Smoking history, *n* (%)	5 (17%)	2 (12%)	3 (23%)	0.4
**Medical History**				
History of MI, *n* (%)	16 (53%)	7 (42%)	9 (69%)	0.2
CHF, *n* (%)	15 (50%)	6 (35%)	9 (69%)	0.2
Urolithiasis, *n* (%)	4 (13%)	1 (6%)	3 (23%)	0.4
CKD at admission, *n* (%)	10 (33%)	3 (18%)	7 (54%) *	0.05
Creatinine level at admission, μmol/L	171 ± 85	181 ± 93	162 ± 78	0.7
GFR at admission, mL/min/1.73 m^2^	37 ± 25	37 ± 22	39 ± 30	0.8
CKD KDIGO (G4-G5)	13 (43%)	7 (41%)	6 (46%)	0.8
Urine volume < 0.5 mL/kg/h for 6 h	22 (73%)	12 (70%)	10 (77%)	0.2
**Angiography data, lesions > 70%**				
LAD, *n* (%)	16 (53%)	7 (42%)	9 (69%)	0.6
LCA, *n* (%)	14 (47%)	6 (35%)	8 (61%)	0.9
RCA, *n* (%)	13 (43%)	8 (47%)	5 (38%)	0.8
**Complications following MI**				
AHF at admission FC > I, *n* (%)	22 (73%)	15 (88%)	7 (54%) *	0.04
LV aneurysm, *n* (%)	7 (23%)	4 (23%)	3 (23%)	0.8
Recurrent MI, *n* (%)	8 (27%)	1 (6%)	7 (54%) *	0.02
Postinfarction angina, *n* (%)	9 (30%)	1 (6%)	7 (54%) *	0.009
**Cause of death**				
Cardiogenic shock	25 (86%)	12 (70%)	13 (100%) *	0.04
Cardiac rupture	2 (7%)	2 (28%)	0	0.9
Arrhythmogenic shock (VF)	2 (7%)	1 (6%)	1 (8%)	0.4

Note: *—statistically significant differences between the groups. Abbreviations: AHF—acute heart failure, CAD—coronary artery disease, CHF—chronic heart failure, CKD—chronic kidney disease, FC—functional class, GFR—glomerular filtration rate, MI—myocardial infarction, KDIGO—Kidney Disease: Improving Global Outcomes, LAD—left anterior descending artery, LCA—left circumflex artery, LV—left ventricle, RCA—right coronary artery, STEMI—ST-segment elevation myocardial infarction, VF—ventricular fibrillation.

**Table 2:** Dynamics of cardiac and kidney macrophages in patients (*n* = 30) with fatal MI, depending on the period of MI.

Parameters (Cells)	All Patients (*n* = 30)	Group 1 (*n* = 17)	Group 2 (*n* = 13)	*p* Value
Kidney CD163+	55 (32; 97)	55 (34; 72)	58 (32; 97)	0.8
Cardiac CD163+ (IA)	460 (62; 846)	82 (34; 285)	697 (545; 982) *	0.001
Cardiac CD163+ (peri-IA)	82 (49; 135)	62 (42; 78)	135 (82; 220) *	0.005
Cardiac CD163+ (non-IA)	66 (45; 93)	70 (45; 87)	63 (59; 121)	0.4
Kidney CD206+	4 (2; 6)	6 (5; 8)	2 (1; 2) *	0.00004
Cardiac CD206+ (IA)	31 (12; 106)	21 (12; 43)	99 (31; 249) *	0.02
Cardiac CD206+ (peri-IA)	24 (12; 41)	16 (11; 29)	36 (15; 43)	0.1
Cardiac CD206+ (non-IA)	15 (4; 33)	16 (5; 36)	14 (4; 16)	0.2
Kidney CD68+	30 (23; 51)	30 (24; 49)	35 (23; 51)	0.9
Cardiac CD68+ (IA)	106 (56; 376)	59 (52; 95)	376 (136; 634) *	0.00003
Cardiac CD68+ (peri-IA)	78 (44; 154)	48 (36; 83)	154 (85; 232) *	0.004
Cardiac CD68+ (non-IA)	67 (38; 115)	44 (33; 75)	95 (61; 141) *	0.03
Kidney stabilin-1+	2 (1; 3)	1 (1; 4)	2 (1; 2)	0.8
Cardiac stabilin-1+ (IA)	1,5 (0; 102)	0 (0; 1)	126 (42; 216) *	0.0001
Cardiac stabilin-1+ (peri-IA)	1 (0; 13)	0 (0; 2)	24 (1; 70) *	0.01
Cardiac stabilin-1+ (non-IA)	0 (0; 3)	0 (0; 0)	0 (0; 13)	0.3

Note: *—statistically significant difference between the groups. Abbreviations: IA—infarct area, MI—myocardial infarction.

**Table 3:** Features of macrophages diversity in the kidneys in patients (*n* = 30) with fatal myocardial infarction (MI), depending on the presence of chronic kidney disease (CKD) in history.

Parameters (Cells)	All Patients (*n* = 30)	CKD+ (*n* = 10)	CKD− (*n* = 20)	*p* Value
Kidney CD163+	55 (32; 97)	61 (34; 97)	44 (32;88)	0.3
Kidney CD206+	4 (2; 6)	2 (2; 3)	5 (2; 6) *	0.004
Kidney CD68+	30 (23; 51)	35 (17; 51)	30 (23; 51)	0.7
Kidney stabilin-1+	2 (1; 3)	1 (1; 2)	2 (1; 3)	0.3

Note: *—statistically significant differences between the groups.

**Table 4:** Summary of multiple linear regression of clinical data and the number of CD206+, CD68+, CD163+, and stabilin-1+ cells in the kidney and early fatal outcome in patients with fatal MI (*n* = 30).

Variable	β (Standard Deviation)	T	*p* Value
History of MI	0.6	3.6	0.0002
STEMI	−0.5	−2.9	0.007
Anterior-inferior MI	−0.1	−0.8	0.3
AHF at admission FC > I	0.4	2.5	0.02
Kidney CD206+ cells	−0.4	−3.1	0.005
Kidney CD163+ cells	−0.1	−0.9	0.3
Kidney CD68+ cells	0.04	0.2	0.7
Kidney stabilin-1+ cells	0.2	1.3	0.2

Abbreviations: AHF—acute heart failure, FC—functional class, MI—myocardial infarction, STEMI—ST-segment elevation myocardial infarction.
